# Potential determinants of obesity among children and adolescents in Germany: results from the cross-sectional KiGGS study

**DOI:** 10.1186/1471-2458-9-46

**Published:** 2009-02-02

**Authors:** Christina Kleiser, Angelika Schaffrath Rosario, Gert BM Mensink, Reinhild Prinz-Langenohl, Bärbel-Maria Kurth

**Affiliations:** 1 Department of Epidemiology and Health Reporting, Robert Koch Institute, Seestr. 10, 13353 Berlin, Germany; 2 Department of Nutrition and Food Science, Pathophysiology of the Nutrition, University of Bonn, Endenicher Allee 11-13, 53115 Bonn, Germany

## Abstract

**Background:**

Obesity among children and adolescents is a growing public health problem. The aim of the present paper is to identify potential determinants of obesity and risk groups among 3- to 17-year old children and adolescents to provide a basis for effective prevention strategies.

**Methods:**

Data were collected in the German Health Interview and Examination Survey for Children and Adolescents (KiGGS), a nationally representative and comprehensive data set on health behaviour and health status of German children and adolescents. Body height and weight were measured and body mass index (BMI) was classified according to IOTF cut-off points. Statistical analyses were conducted on 13,450 non-underweight children and adolescents aged 3 to 17 years. The association between overweight, obesity and several potential determinants was analysed for this group as well as for three socio-economic status (SES) groups. A multiple logistic regression model with obesity as the dependent variable was also calculated.

**Results:**

The strongest association with obesity was observed for parental overweight and for low SES. Furthermore, a positive association with both overweight (including obesity) and obesity was seen for maternal smoking during pregnancy, high weight gain during pregnancy (only for mothers of normal weight), high birth weight, and high media consumption. In addition, high intakes of meat and sausages, total beverages, water and tea, total food and beverages, as well as energy-providing food and beverages were significantly associated with overweight as well as with obesity. Long sleep time was negatively associated with obesity among 3- to 10-year olds. Determinants of obesity occurred more often among children and adolescents with low SES.

**Conclusion:**

Parental overweight and a low SES are major potential determinants of obesity. Families with these characteristics should be focused on in obesity prevention.

## Background

The increased prevalence of overweight and obesity among children and adolescents is a severe public health problem across the developed and the developing world [1]. Nationally representative data from the German Health Interview and Examination Survey for Children and Adolescents (KiGGS) show that in Germany, currently 15% of the 3- to 17-year olds are considered overweight or obese according to the national reference. The prevalence of obesity is 6.3% [2]. This implies an increase of 50% in the prevalence of overweight compared to the early 1990s, while the prevalence of obesity has doubled. According to IOTF cut-off points, 4.9% of the 3- to 17-year olds are obese and 18.8% are overweight (or obese).

Obesity is the consequence of a long-term imbalance between energy intake and energy expenditure, determined by food intake and physical activity and influenced by biological and environmental factors. Potential risk factors for obesity in early life include genetic, physical, lifestyle, and environmental conditions [3-12]. These factors affect energy balance on different levels and may interact. Therefore, the particular causal pathways involved remain unclear to a certain extent.

Obesity may have several short-term consequences (e.g. social discrimination, lower quality of life, increased cardiovascular risk factors, diseases like asthma) [13] and long-term consequences (e.g. persistence of obesity, increased morbidity, a higher prevalence of cardiovascular risk factors in adulthood) [4,13] and causes an important economic burden [14]. Obesity should therefore be prevented as early as possible. For establishing effective intervention, it is important to identify major determinants in an early stage of life. For Germany, previous data were predominantly derived from school entrance health examinations and therefore included only a limited age range. For the first time, comprehensive and nationally representative data for the entire group of children and adolescents living in Germany are now available with the KiGGS study. The aim of the present paper is to identify major potential determinants of obesity and risk groups among 3- to 17-year olds.

## Methods

### Sample

From May 2003 to May 2006, a total of 17,641 children and adolescents aged 0 to 17 years participated in KiGGS. The sample was derived from 167 sample points (communities) representative for Germany, stratified by federal state and community type. Within each sample point, participants were selected at random from the official registers of local residents with stratification in one-year age groups. The overall response rate was 66.6% [15].

The examination took place in examination centres at the sample points. Information about socio-demographic characteristics, living conditions, health, and health behaviour was obtained with self-administered questionnaires filled in by the parents. Some information was additionally filled in by participants aged 11 years or older. For migrants with only limited knowledge of the German language, a shortened version was available in various languages [16]. Participants beyond 14 years of age and all parents provided written informed consent prior to the interview and examination. The survey was approved by the Federal Office for Data Protection and by the Charité-Universitätsmedizin Berlin ethics committee.

The present analyses are restricted to children and adolescents aged 3 to 17 years (n = 14,836). 89 participants with no information on weight status and 1,297 underweight participants (thinness grade 1, defined by Cole et al. [17]) were excluded. The reason for this exclusion is a presumed difference of socio-economic and behavioural determinants between underweight and overweight people [18]. A sensitivity analysis without this exclusion only marginally changed our results. The sample thus comprised 13,450 participants. For some analyses, the number of participants was smaller due to missing data. Food intake data was available for 12,792 children and adolescents. The analyses concerning socio-economic status (SES) included 13,102 participants. For the multivariable model 10,021 children and adolescents with complete data were included.

### Data obtained from the physical examination

Body height was measured, without wearing shoes, by trained staff with an accuracy of 0.1 cm, using a portable Harpenden stadiometer (Holtain Ltd., Crymych, UK). Body weight was measured with an accuracy of 0.1 kg, wearing underwear, with a calibrated electronic scale (SECA, Birmingham, UK). Body mass index (BMI) in kg/m^2 ^was classified according to International Obesity Task Force (IOTF) cut-off points for children and adolescents [19]. Throughout this paper, the term overweight always includes obesity. The term obesity is restricted to exclusively obese persons as defined by the IOTF.

Furthermore, 10- to 17-year olds self-assessed their pubic hair status on the basis of Tanner-drawings [20]. They were classified into three categories: pre-pubertal (Tanner stage 1), pubertal (Tanner stage 2 or 3) and post-pubertal (Tanner stage 4 to 6).

### Data obtained from parental questionnaires for all ages

Information on parents' income, occupational status and education was used to quantify the SES which was categorised into low, medium, and high SES [21,22]. A participant was defined to have a one-sided/two-sided migration background if one/both of the parents were not born in Germany and/or have no German citizenship [16]. Self-reported height and weight of mothers and fathers were used to calculate parental BMI which was classified into overweight (including obesity) or non-overweight according to the WHO cut-off points of 25 kg/m^2 ^[23]. We considered the weight status of the biological parents only. A separate category "incomplete data" was used in the analysis; otherwise all single-parent families would have been excluded. Parental smoking at the time of interview was documented for mothers and fathers. Maternal smoking during pregnancy was classified into yes or no. Furthermore, reported birth weight (in grams) was defined as low when less than 2500 g [24] and high when more than 4000 g. In addition, mothers were asked for weight gain during pregnancy (in kg), the presence of maternal diabetes during pregnancy and breastfeeding behaviour.

### Data obtained from parental questionnaires for 3- to 10-year olds

Physical activity was obtained as the frequency of doing sports (separately for within or outside a sports club) in categories of almost daily/3–5 times per week/1–2 times per week/seldom/never. Media consumption was assessed as the average time daily spent on watching television/video and using the computer in categories of not at all/about 30 minutes/1–2 hours/3–4 hours/more than 4 hours and was documented separately for weekdays and the weekend. Sleep duration was reported as the average hours of sleep per day. Food consumption was assessed with a self-administered semi-quantitative food frequency questionnaire (FFQ) including 54 food items, which was completed at home. The FFQ was developed consulting several experts with experience in children's dietary assessment to include the most relevant food groups for children and adolescents. A detailed description is presented elsewhere [25].

### Data obtained from questionnaires for 11- to 17-year olds

Self-reported vigorous physical activity ("During leisure time, how often are you physically active in a way that makes you out of breath or makes you sweat?") was assessed in categories of almost daily/3–5 times per week/1–2 times per week/seldom/never. Media consumption was asked in a similar way as for younger participants. However, no difference between weekdays and weekends was made. Additionally, playing videogames was asked. Sleep duration was obtained as hours of sleep during the last night. Food consumption was obtained with the same FFQ as mentioned above. Risk groups with symptoms of possible eating disorders were determined with the SCOFF questionnaire (SCOFF is an acronym reflecting the five questions addressing core features of eating disorders) [26,27]. Furthermore, smoking behaviour and alcohol consumption was asked. Regular alcohol consumers were defined as adolescents drinking at least one glass of alcohol (beer, wine, liquor) per week.

### Use of variables in the analysis

For all participants, physical activity was recalculated as times per week and additionally categorised into approximate age-specific tertiles (approximate because of the ordinal scale of the initial variables). Total media consumption was calculated in hours per day for use as continuous variable and additionally categorised into age-specific tertiles. Reported hours of sleeping time was used to construct a sleeping score (expressed as midranks ranging from 0 to 100%), which can be interpreted as percentiles as described by Bayer et al. [28]. It was used in tertiles as well as continuously (per 20% increase in midranks). Furthermore, age – and sex-specific tertiles of food intake were calculated for several food groups. The total intake of energy-providing food and beverages was used as an overall indicator of food intake.

### Statistical analysis

First, frequencies of overweight (including obesity) as well as frequencies of obesity stratified by potential determinants were analysed. The corresponding odds ratios (OR), adjusted for age and gender, were calculated with binary logistic regression models (univariable analysis). Second, frequencies of overweight as well as frequencies of obesity were compared across the tertiles of food intake. Binary logistic regression models were calculated, adjusted for age and gender, and the p-values for the comparison of the highest vs. the lowest tertile are reported. Third, a multivariable binary logistic regression analysis was performed with obesity as the dependent variable. All variables which showed a statistically significant association with obesity in the univariable analysis were included as well as statistically significant qualitative interactions with gender, age or parental overweight. An interaction was considered qualitative when the interaction term was statistically significant in the univariable analysis, and when the interaction remained qualitative in the multivariable model in the sense that there was a statistically significant association with obesity in one group, but not in the other. In the multivariable model media consumption, physical activity, sleep duration, and food intake were included as continuous variables and all others as class variables. Total intake of energy-providing food and beverages (in units of 100 g per day) was used as an overall indicator for total food intake. Since intake of energy-providing food and beverages was far from significant (p = 0.9) and the estimators for all other variables changed only marginally, this variable was not included in the final model. Furthermore, pubertal stage (which is strongly associated with age) and SCOFF were not included in the multivariable model. These characteristics may be effects rather than causes of obesity. Furthermore, these data are only available for older participants. Finally, a descriptive analysis separately for the three SES groups was performed. First, the distribution of the potential determinants by SES group was described. Then, the frequency of obesity was tabulated in subgroups defined by SES and the potential determinants, for those variables which showed a significant univariable interaction with SES.

All analyses used sampling weights [15] and the survey procedures of SAS version 9.1 [29] to take the cluster structure of the multi-stage sample into account. A p-value < 0.05 (in interaction analyses p < 0.10) was considered to be statistically significant.

## Results

### Univariable analysis

The associations between relative weight status and different social, environmental and behavioural determinants, adjusted for age and gender, are shown in Table 1. Low SES is associated with higher frequencies of overweight (including obesity) as well as with obesity. Children and adolescents with a two-parent migration background are more often overweight and also more often obese than non-migrants. However, this does not apply to children and adolescents with a one-parent migration background. Children and adolescents whose parents are overweight, whose parents smoke, whose mother smoked during pregnancy, whose mother gained weight more than 20 kg during pregnancy, who were not ever predominantly breastfed, who had high birth weight, who have a post-pubertal status, a low level of physical activity, high media consumption, who eat most energy-providing food and beverages and who show symptoms of eating disorders are more often overweight and more often obese than their respective counterparts (Table 1). Low birth weight is statistically significantly associated with a higher proportion of obesity, but not with overweight (data not shown). Diabetes during pregnancy, the presence of siblings and smoking of the adolescents (11 to 17 years) are associated with a higher proportion of overweight, but not with obesity (data not shown). There are no statistically significant associations between overweight or obesity and gender, living in Eastern vs. Western Germany, community size, and regular alcohol consumption of the adolescents (data not shown).

**Table 1:** Frequency of overweight (including obesity) and obesity according to potential determinants [% (95% CI)] and odds ratio

	N^a)^	Overweight^b) ^(including obesity) [%]	OR^c) ^for Overweight (95% CI)	Obesity^b) ^[%]	OR^c) ^for Obesity (95% CI)
**Personal and social factors**					

Socio-economic status					
Low	3655	26.6	2.12 (1.8–2.4)	8.9	3.76 (3.0–4.7)
Medium	6121	20.3	1.47 (1.3–1.7)	4.7	1.87 (1.4–2.5)
High	3326	14.5	ref.	2.5	ref.
Missing data	348				
p-value*			<0.001		<0.001

Migration background					
One-parent	920	19.0	1.00 (0.8–1.2)	4.7	1.01 (0.7–1.5)
Two-parent	2031	25.2	1.38 (1.2–1.6)	7.6	1.61 (1.3–2.0)
Non-Migrant	10444	19.7	ref.	4.9	ref.
Missing data	55				
p-value*			<0.001		<0.001

Parental overweight at time of interview (biological parents)					
Both overweight/obese	2696	32.4	4.92 (4.1–6.0)	10.6	10.2 (6.7–15.3)
Mother overweight/obese	1056	18.5	2.36 (1.8–3.1)	4.4	4.01 (2.4–6.7)
Father overweight/obese	3435	17.5	2.21 (1.8–2.7)	3.6	3.27 (2.1–5.1)
None overweight/obese	2707	8.6	ref.	1.1	ref.
Incomplete data^d)^	3556	24.4	3.19 (2.6–3.9)	6.5	5.76 (3.7–8.9)
p-value*			<0.001		<0.001

Parental smoking (at time of interview)					
Father and mother smoke	2534	27.8	1.91 (1.7–2.2)	8.8	2.46 (1.9–3.1)
Only mother smokes^e)^	1788	24.3	1.52 (1.3–1.8)	6.7	1.76 (1.3–2.4)
Only father smokes	2474	19.7	1.20 (1.1–1.4)	4.8	1.28 (1.0–1.7)
Neither mother nor father smokes	6340	17.0	ref.	3.8	ref.
Missing data	314				
p-value*			<0.001		<0.001

Pubertal stage (10–17 years)					
Pre-pubertal	904	23.0	ref.	3.9	ref.
Pubertal	1633	23.7	1.16 (0.9–1.5)	5.4	1.60 (0.9–2.8)
Post-pubertal	4497	23.6	1.72 (1.3–2.3)	6.6	2.35 (1.2–4.5)
Not assessed (3–9 years) or missing data	6416				
p-value*			<0.001		0.031

SCOFF (11–17 years)					
Conspicuous	1436	40.7	3.42 (2.9–4.0)	14.1	4.61 (3.6–5.8)
Inconspicuous	4663	18.0	ref.	3.8	ref.
Not assessed (3–10 years) or missing data	7351				
p-value			<0.001		<0.001

**Early life factors**					

Maternal smoking during pregnancy					
Yes	2273	27.8	1.68 (1.5–1.9)	8.4	1.93 (1.6–2.4)
No	10724	18.9	ref.	4.6	ref.
Missing data	453				
p-value			<0.001		<0.001

Weight gain during pregnancy					
Up to 20 kg	10748	19.5	ref.	5.0	ref.
21 kg and more	994	28.3	1.77 (1.5–2.1)	8.5	1.92 (1.4–2.6)
Missing data	1708				
p-value			<0.001		<0.001

High birth weight					
Yes (>4000 g)	1451	28.4	1.73 (1.5–2.0)	8.4	1.83 (1.5–2.3)
No	11342	19.3	ref.	4.9	ref.
Missing data	657				
p-value			<0.001		<0.001

Ever predominantly breastfed					
Yes	7999	17.9	ref.	4.2	ref.
No	3977	25.3	1.50 (1.3–1.7)	7.3	1.74 (1.4–2.2)
Missing data	1474				
p-value			<0.001		<0.001

**Behavioural factors**					

Sleep duration					
Lowest tertile	4451	22.5	1.24 (1.1–1.4)	5.9	1.24 (1.0–1.6)
Middle tertile	4399	19.4	0.94 (0.8–1.1)	5.0	0.90 (0.7–1.1)
Highest tertile	4318	19.6	ref.	4.9	ref.
Missing data	282				
p-value*			<0.001		0.01

Media consumption					
Lowest tertile	4293	15.6	ref.	3.7	ref.
Middle tertile	4076	20.0	1.34 (1.2–1.5)	4.7	1.24 (1.0–1.6)
Highest tertile	4326	26.1	1.95 (1.7–2.2)	7.3	2.06 (1.6–2.6)
Missing data	755				
p-value*			<0.001		<0.001

Physical activity					
Lowest tertile	4479	23.1	1.40 (1.2–1.6)	6.4	1.41 (1.1–1.8)
Middle tertile	4364	20.0	1.17 (1.0–1.3)	4.7	1.03 (0.8–1.3)
Highest tertile	4035	18.2	ref.	4.7	ref.
Missing data	572				
p-value*			<0.001		<0.01

Intake of energy-providing food and beverages					
Lowest tertile	3691	19.4	ref.	4.9	ref.
Middle tertile	3773	20.5	1.08 (0.9–1.2)	4.7	0.95 (0.7–1.2)
Highest tertile	3791	22.5	1.22 (1.1–1.4)	6.4	1.32 (1.1–1.7)
Missing data	2195				
p-value*			0.006		0.011

N = 13,450 3- to 17-year olds (after exclusion of underweight participants)

*p for trend

^a) ^unweighted

^b) ^Based on IOTF cut points

^c) ^Odds ratio, adjusted for age and gender

^d) ^Children not living with both biological parents or for which information on the mother's and/or father's BMI is missing

^e) ^Father doesn't smoke or no information on the father available.

**Figure 1:**
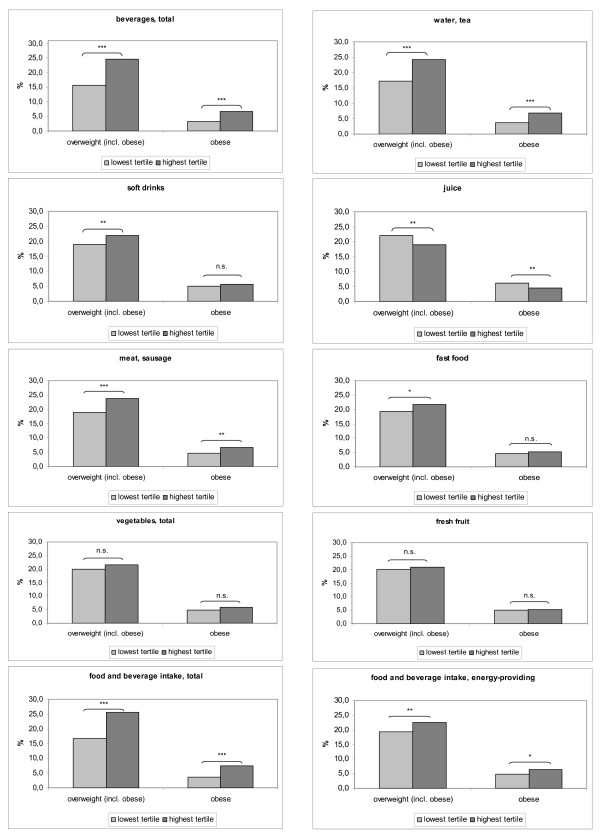
**Association of food intake and weight status**. Frequency of overweight (including obesity) and obesity by lowest and highest tertiles of intake of selected food groups. N = 12,792 3- to 17- year olds (underweight participants excluded). p-value for lowest vs. highest tertile from univariable regression models with overweight or obesity as independent variable, adjusted for age and gender. n.s. = not significant; * = p < 0.05; ** = p < 0.01;*** = p < 0.001.

Results of the analysis of weight status and dietary intake (lowest vs. highest tertile of food intake) are shown in Figure 1. There is a statistically significant positive association between overweight (including obesity) as well as obesity and the total beverage intake, the consumption of water (including tea), of meat and sausages, the total food and beverage intake, and the intake of energy-providing food and beverages. Furthermore, overweight is positively associated with the consumption of soft drinks and fast food. There is a statistically significant negative association between both overweight and obesity and the consumption of juice, as well as between overweight and salty snacks and butter/margarine (data not shown). No association appears between weight status and the consumption of vegetables and fresh fruit (Figure 1) as well as for pasta/rice/potatoes, bread/cereals, milk/dairy products, fish, eggs, and sweets (data not shown).

### Multivariable analysis

The multivariable logistic regression model (Table 2) contains three significant qualitative interactions: the interaction of age with sleep duration and migration background, and the interaction of maternal weight status and a high weight gain during pregnancy. The model shows a statistically significant positive association between obesity and low SES, parental overweight, maternal smoking during pregnancy, high birth weight and media consumption. Furthermore, migration background among 3- to 13-year olds and weight gain during pregnancy more than 20 kg (when the mother is of normal weight) are statistically significantly associated with obesity. There is a negative association with obesity for sleep duration among 3- to 10-year olds. Parental overweight shows the strongest association with obesity. The OR for obesity was 11.2 when both parents are overweight, compared to children with no overweight parents (with weight gain during pregnancy <= 20 kg). The OR for obesity when only the mother (father) is overweight is 4.3 (3.5). Children and adolescents with low SES have a more than two times higher OR for obesity than those with high SES. No statistically significant association with obesity is seen for parental smoking at the time of interview, breastfeeding and physical activity. Furthermore, migration background among 14- to 17-year olds and sleep duration among 11- to 17-year olds are not statistically significantly associated with obesity.

**Table 2:** Results of the multivariable logistic regression model with obesity as dependent variable, adjusted for age and gender

	Odds ratio	95%-CI	p
Socio-economic status			
Low	2.26	(1.6–3.2)	
Medium	1.49	(1.0–2.1)	
High	ref.	-	<0.001

Migration background (interaction with age)			
Migrant vs. Non-migrant^a)^, in 3- to 13-year olds	1.66	(1.1–2.4)	0.0105
Migrant vs. Non-migrant^a)^, in 14- to 17-year olds	0.67	(0.3–1.3)	0.2227

Parental overweight at time of interview (biological parents) (interaction with weight gain during pregnancy)			
*With weight gain during pregnancy <= 20 kg:*			
Both overweight/obese	11.24	(6.4–19.7)	
Mother overweight/obese	4.30	(2.2–8.6)	
Father overweight/obese	3.54	(2.0–6.3)	
None overweight/obese	ref.	-	
Incomplete data^b)^	4.75	(2.6–8.5)	<0.001
*With weight gain during pregnancy > 20 kg:*			
Both overweight/obese	2.83	(1.0–7.7)	
Mother overweight/obese	1.08	(0.4–3.1)	
Father overweight/obese	3.54	(2.0–6.3)	
None overweight/obese	ref.	-	
Incomplete data^b)^	2.23	(0.9–5.7)	<0.001

Parental smoking (at time of interview)			
Mother and/or father	1.30	(1.0–1.7)	0.0601
None	ref.	-	

Maternal smoking during pregnancy			
Yes	1.37	(1.0–1.8)	0.0267
No	ref.	-	

Weight gain during pregnancy (interaction with mother's current relative weight status)			
Mother normal weight: Weight gain during pregnancy > 20 kg vs. <= 20 kg	2.81	(1.6–5.0)	0.0004
Mother overweight: Weight gain during pregnancy > 20 kg vs. <= 20 kg	0.71	(0.3–1.6)	0.3971
Incomplete data^b)^: Weight gain during pregnancy > 20 kg vs. <= 20 kg	1.32	(0.7–2.5)	0.4081

High birth weight			
Yes (> 4000 g)	1.87	(1.4–2.5)	<0.001
No	ref.	-	

Ever predominantly breastfed			
Yes	0.87	(0.7–1.1)	0.2970
No	ref.	-	

Sleep duration per 20% increase in midranks (interaction with age)			
Sleep duration in 3- to 10-year olds	0.89	(0.8–1.0)	0.0461
Sleep duration in 11- to 17-year olds	0.99	(0.9–1.1)	0.8498

Media consumption (hours per day)	1.09	(1.0–1.2)	0.0107

Physical activity (times per week)	0.98	(0.9–1.0)	0.4514

N = 10,021 3- to 17-year olds (after exclusion of underweight participants and those with missing data)

^a) ^Including children with a one-parent migration background

^b) ^Children not living with both biological parents or for which information on the mother's and/or father's BMI is missing.

### Associations with SES

As shown in Table 3, most presented determinants are more common among the group with low SES, compared to those with medium or high SES. The exceptions are a one-parent migration background, pubertal stage, and high birth weight, which show a similar distribution in all SES groups. Parents of children and adolescents with low SES are more often overweight. This is also true when the participants with incomplete data on parental weight among the low SES group (which is mostly due to single-parent families) are excluded.

**Table 3:** Distribution of potential determinants, differentiated by SES [% (95% CI)]

	Low SES N^a) ^= 3655	Medium SES N^a) ^= 6121	High SES N^a) ^= 3326
Migration background			
One-parent	8.0 (6.8–9.2)	7.2 (6.3–8.1)	9.0 (7.6–10.4)
Two-parent	31.0 (27.3–34.7)	12.5 (10.7–14.3)	6.0 (4.8–7.2)
Non-Migrant	61.0 (56.8–65.2)	80.3 (78.0–82.5)	85.0 (82.9–87.1)

Parental overweight at time of interview (biological parents)			
Both overweight/obese	24.1 (22.4–25.9)	21.9 (20.5–23.3)	14.9 (13.4–16.3)
Mother overweight/obese	8.3 (7.3–9.4)	8.8 (7.8–9.7)	6.9 (6.0–7.9)
Father overweight/obese	19.2 (17.6–20.7)	27.5 (26.0–29.0)	31.3 (29.6–33.1)
None overweight/obese	11.3 (10.0–12.6)	20.0 (18.7–21.3)	32.4 (30.5–34.4)
Incomplete data^b)^	37.1 (34.8–39.3)	21.9 (20.3–23.4)	14.5 (13.0–16.0)

Parental smoking (at time of interview)			
Father and mother smoke	31.0 (28.8–33.3)	18.7 (17.5–19.9)	11.2 (9.9–12.6)
Only mother smokes^c)^	10.8 (9.6–12.1)	11.6 (10.7–12.6)	8.4 (7.3–9.6)
Only father smokes	23.5 (21.6–25.3)	20.2 (19.0–21.5)	15.1 (13.5–16.7)
Neither mother nor father smokes	34.6 (32.1–37.2)	49.4 (47.7–51.1)	65.2 (63.1–67.3)

Pubertal stage (10–17 years)			
Pre-pubertal	10.5 (8.9–12.0)	11.5 (10.2–12.9)	11.4 (9.8–12.9)
Pubertal	22.1 (20.3–23.9)	20.8 (19.3–22.3)	19.6 (17.6–21.7)
Post-pubertal	67. 4 (65.4–69.5)	67.6 (65.8–69.4)	69.0 (66.5–71.5)

SCOFF (11–17 years)			
Conspicuous	29.1 (26.7–31.5)	22.5 (20.7–24.3)	16.7 (14.6–18.8)
Inconspicuous	70.9 (68.5–73.3)	77.5 (75.7–79.3)	83.3 (81.2–85.4)

Maternal smoking during pregnancy			
Yes	30.4 (28.4–32.4)	16.6 (15.4–17.9)	8.3 (7.1–9.5)
No	69.6 (67.5–71.6)	83.4 (82.1–84.6)	91.7 (90.5–92.9)

High birth weight			
Yes (> 4000 g)	10.6 (9.4–11.8)	11.3 (10.2–12.3)	11.8 (10.6–12.9)
No	89.4 (88.2–90.6)	88.7 (87.7–89.8)	88.2 (87.1–89.4)

Ever predominantly breastfed			
Yes	53.6 (51.0–56.2)	65.9 (64.0–67.9)	78.7 (76.9–80.6)
No	46.4 (43.8–49.0)	34.1 (32.1–36.0)	21.3 (19.4–23.1)

Media consumption			
Lowest tertile	24.0 (22.2–25.8)	33.3 (31.7–35.0)	48.2 (45.3–51.1)
Middle tertile	32.3 (30.3–34.3)	33.4 (31.9–34.9)	32.2 (29.9–34.6)
Highest tertile	43.7 (41.4–46.0)	33.3 (31.5–35.2)	19.6 (17.7–21.4)

Physical activity			
Lowest tertile	42.5 (40.6–44.3)	34.8 (33.2–36.4)	26.8 (24.8–28.7)
Middle tertile	29.7 (27.9–31.5)	34.2 (32.9–35.5)	37.6 (35.6–39.6)
Highest tertile	27.9 (26.0–29.7)	31.0 (29.5–32.6)	35.7 (33.6–37.8)

Intake of energy-providing food and beverages			
Lowest tertile	30.4 (28.2–32.5)	35.6 (33.8–37.3)	36.3 (34.3–38.3)
Middle tertile	28.6 (26.9–30.3)	34.2 (32.6–35.8)	37.8 (36.0–39.6)
Highest tertile	41.0 (38.9–43.2)	30.2 (28.5–32.0)	25.9 (24.0–27.8)

N = 13,102 3- to 17-year olds (after exclusion of underweight participants and those with missing data in SES)

^a) ^unweighted

^b) ^Children not living with both biological parents or for which information on the mother's and/or father's BMI is missing

^c) ^Father doesn't smoke or no information on the father available.

Table 4 shows the frequency of obesity according to potential determinants, differentiated by SES groups. Included are only potential determinants which show a statistically significant univariable interaction with SES. The highest frequency of obesity is found among children and adolescents with low SES of which both parents are overweight (12.4%). With the exception of parental overweight, our data show that children and adolescents with low SES, even if they have favourable levels of other potential determinants, are more often obese than those with medium or high SES, even if the latter have unfavourable levels of those determinants. As an example, the frequency of obesity among those with low SES but also low media consumption is higher than among those with medium or high SES and high media consumption.

**Table 4:** Frequency of obesity according to potential determinants, differentiated by SES [% (95% CI)]

	Low SES N^a) ^= 3655	Medium SES N^a) ^= 6121	High SES N^a) ^= 3326
Parental overweight at time of interview (biological parents)			
Both overweight/obese	12.4 (10.1–14.7)	10.6 (8.6–12.5)	7.3 (4.9–9.8)
Mother overweight/obese	8.9 (4.7–13.2)	2.6 (0.9–4.4)	2.7 (0.2–5.1)
Father overweight/obese	5.6 (3.4–7.8)	3.4 (2.4–4.5)	2.7 (1.7–3.6)
None overweight/obese	3.6 (1.7–5.5)	0.8 (0.3–1.4)	0.5 (0.1–0.9)
Incomplete data^b)^	10.0 (8.2–11.8)	4.8 (3.6–6.0)	1.9 (0.6–3.2)

Ever predominantly breastfed			
Yes	8.4 (7.0–9.9)	4.0 (3.2–4.7)	1.8 (1.2–2.4)
No	9.7 (7.9–11.5)	6.4 (4.9–8.0)	4.7 (3.0–6.3)

Media consumption			
Low	9.6 (7.2–12.1)	2.7 (1.9–3.4)	1.7 (1.1–2.4)
Middle	7.3 (5.6–9.1)	4.3 (3.3–5.4)	2.7 (1.7–3.8)
High	9.1 (7.4–10.9)	7.0 (5.7–8.3)	3.8 (2.2–5.5)

N = 13,102 3- to 17-year olds (after exclusion of underweight participants and those with missing data in SES)

^a) ^unweighted

^b) ^Children not living with both biological parents or for which information on the mother's and/or father's BMI is missing.

## Discussion

### Main findings

Our data confirm associations with overweight and obesity for many of the supposed determinants. Independently of other factors, a positive association was observed between obesity and low SES, migration background (up to age 13), parental overweight, high weight gain during pregnancy (when the mother is of normal weight), maternal smoking during pregnancy, high birth weight, and high media consumption, as well as a negative association with sleep duration for 3- to 10-year olds. The observed univariable associations with parental smoking at the time of interview, breastfeeding, physical activity, and food intake did not significantly contribute to the multiple logistic regression model.

### Personal and social aspects

Consistent with other studies [6,30], the strongest determinant in the multivariable analysis was parental overweight. When both parents are overweight, the risk of obesity for the offspring was increased 11-fold (in case of a low weight gain during pregnancy). If only one parent is overweight, the OR was still higher than that for any other determinant in the model. The ORs changed only by approximately 10% when the analysis was extended to include all parents, biological as well as social ones. The strong association with parental overweight may be explained by genetic, as well as environmental and behavioural factors [6,31,32]. A recent twin-study [31] concludes that genetic factors play the most important role in determining which children become obese in a changed environment. The secular increase in obesity rates, however, cannot be explained by genetic variation, but is an example for gene-environment interactions. A possible infectious origin of obesity [33] or exposure to environmental contaminants such as endocrine disruptors, which have been alleged to be a possible cause of overweight [34], would also be expected to cluster within families.

The group with incomplete data on parental overweight shows a higher obesity risk than those with none or just one overweight parent. This is probably due to the fact that single-parent families more often have a low SES. This in turn might be due to less income and the difficulty to manage job and family, especially for single mothers. Furthermore, there might be a tendency among overweight parents not to report their weight.

Almost all analysed potential determinants of obesity were more prevalent among children and adolescents with low SES. Furthermore, obesity occurred significantly more often among the low SES group, even among those with favourable behaviours, compared to those with medium or high SES and unfavourable behaviours. Up to age 13, children with a two-parent migration background showed a higher obesity risk than those with a one-parent or no migration background. It may be that this difference disappears at higher ages because adolescents behave more similar to native Germans than younger migrants, or because of a cohort effect, or because of different participation patterns among adolescent migrants. Migrants were more often obese than non-migrants within every SES group (data not shown). Similar results have been found for some ethnic minorities in the United States [35,36]. Although SES explains some of the impact of the migration background (and vice versa), migration background remains an independent determinant which also reflects culturally determined attitudes and behaviours [37,38].

We observed an association between pubertal stage and obesity only on the univariable level. The direction of the relationship between weight status and the onset of puberty remains unclear. It has been suggested that obesity can cause an earlier onset of puberty, at least among girls [39,40]. One explanation is that leptin provides the link between body fat and the onset of puberty by affecting gonadotropin secretion [40].

Furthermore, a univariable association of obesity with symptoms of eating disorders has been found. This highlights the importance of taking psychological factors into account when tackling the obesity problem and it reminds one that prevention and intervention measures must take care not to add to the psycho-social burden of obesity.

### Early life aspects

Early childhood is increasingly seen as a critical period for the development of obesity [9]. A combination of certain risk factors may account for an important proportion of obese children [41]. The importance of maternal smoking during pregnancy, high weight gain during pregnancy, and high birth weight observed in our study was also seen in other studies [3,6,30,41]. Our data show a significant interaction between high weight gain during pregnancy and maternal overweight, as was first noticed in a parallel analysis of this dataset [42]. Among overweight mothers, high weight gain during pregnancy was not associated with obesity in the offspring. The association between weight gain in pregnancy and obesity in the offspring might be mediated by high birth weight, and the mediation effect might be different between normal weight and overweight mothers. We ran an additional analysis without high birth weight (data not shown), but the odds ratios changed by less than 10%, so this cannot be the explanation for the interaction effect. Weight gain in pregnancy has been found to increase with maternal BMI, but with a higher variability in overweight women and a decrease in mean weight gain in obese as compared to overweight (but not obese) women [43]. A potential explanation for the interaction effect could be that changes in the intrauterine environment in overweight mothers are similar to the changes occurring with high weight gain during pregnancy. Therefore, the coexistence of both factors may confer no additional increase in obesity risk in the offspring.

High birth weight is an independent risk factor for obesity in our analysis. The OR changed only marginally in the multivariable model. Birth weight is a crude indicator of prenatal growth. The metabolic programming during gestation as well as the foetal environment may play an important role for the association between birth weight and obesity in later life [44].

Recently, it has been suggested that paternal smoking is a risk factor for childhood obesity almost similar in magnitude to smoking of the mother [45]. This may question the causality of the association between maternal smoking in pregnancy and obesity. The variable "mother or father smokes at the time of interview" was used in addition to smoking in pregnancy in the present multivariable model. Parental smoking at the time of interview was only marginally significant; however, when restricted to daily smokers, it remained significant in the multivariable model (data not shown). Hence, smoking of the parents is a marker for families with a higher obesity risk, especially when both parents smoke regularly.

A recent review concluded that breastfeeding seems to have a small protective effect against obesity in later life [3]. This association was not confirmed in the multivariable analysis of the present study. A large randomized intervention trial recently found no effect of breastfeeding on adiposity in 6-year olds [46]. Thus, the positive effects of breastfeeding found in observational studies could be partly due to uncontrolled confounding or selection bias.

### Behavioural aspects

As Swinburn et al. [47] conclude there is a convincing positive association between obesity and sedentary lifestyle, high intake of energy-dense food and a convincing negative relationship with regular physical activity and a high intake of non-starch polysaccharides. Furthermore, increasing aerobic physical activity has been found to be effective in preventing childhood obesity and overweight [48]. In our study, some differences in food intake were found between children and adolescent with different weight status, but the results are not conclusive. Physical activity was only associated with obesity in the univariable analysis. This may be mainly due to the fact that physical activity is only marginally assessed. A major problem in correlating weight status and physical activity as well as food intake is the inaccurate measurements in large-scale epidemiologic studies. Instruments for measuring dietary intake and physical activity are often too crude to draw exact conclusions about energy intake and expenditure. This also applies to the KiGGS study. For long-term weight gain, a relatively small positive energy balance, too small to detect with the usual methods, is sufficient. Furthermore, with cross-sectional studies it is not possible to measure such long-term discrepancies in energy balance. However, longitudinal studies may detect the association between energy imbalance and body fat mass [49]. Furthermore, obese people tend to underreport their food intake more than lean people [50] and also the eating behaviour in the past could be more important than the current food intake.

In the present study, children and adolescent with high media consumption are more often obese than those with lower media consumption time. Media consumption time, as a measure of sedentary behaviour, might be easier to assess than total physical activity, especially in children. When TV watching in hours per day is considered independently from other media consumption in the multivariable model, the OR was slightly higher (OR = 1.14, data not shown) compared to the OR for total media consumption. This was also seen in a recent study among Spanish adolescents [51]. However, the observed impact of media time per hour is small and the causal direction remains unclear.

We observed a negative association between duration of sleep and obesity among 3- to 10-year olds, but not among 11- to 17-year olds. Reviews have also found a stronger association of obesity with sleep duration in younger children, at least when compared to adults [52-54]. However, it is not yet known whether interventions regarding sleep duration are feasible [52]. The interaction with age in our data could also be due to the fact that in 11- to 17-year olds, only sleep duration in the last night was documented, not average sleep duration.

### Strengths and weaknesses

For the first time in Germany, nationally representative data including comprehensive information about health status and health behaviour over the entire age range of children and adolescents are available in a large sample. This allowed us to conduct analyses broad in scope on possible determinants of obesity. This underlines the complexity of obesity aetiology. However, several potential risk factors e.g. early adiposity rebound, catch-up growth, weight gain within the first year, energy intake, were not considered in the present study, since these data were not available. BMI was used to define overweight and obesity instead of excess body fat. In such a large epidemiologic study an accurate measure of total body fat would be very costly. In KiGGS waist and hip circumference (but only for 11- to 17-year olds) as well as triceps and subscapular skinfold thicknesses were measured. The latter, however, were not considered to be more appropriate to estimate total body fat than BMI. The choice of adiposity cut-offs according to IOTF criteria might have reduced the power to detect some associations since the number of obesity cases is rather small using this definition. Another weakness is the method used to assess physical activity. It only gives limited information on physical activity during leisure time, but not on total physical activity including transport, physical activity classes at school etc. Additionally, a relatively rough instrument to measure food consumption (FFQ) was used. Furthermore, because of the cross-sectional nature of our study and the interdependency of many of the variables, no definite statement on causality or causal directions can be made. In future, KiGGS will become a cohort study which may contribute to a better understanding of the cause-effect relationships.

## Conclusion

The major potential determinants for obesity found in the KiGGS study are low SES and parental overweight. Furthermore, low SES is associated with a higher occurrence of most potential determinants of childhood obesity. Therefore, children and adolescents from families with low SES are important groups to focus on in prevention efforts. To reach those people who need support, family-based and low-threshold interventions are important and the complexity of obesity should be kept in mind.

## Abbreviations

BMI: Body mass index; CI: Confidence interval; FFQ: Food frequency questionnaire; IOTF: International Obesity Task Force; OR: Odds ratio; SES: Socio-economic status; WHO: World Health Organisation

## Competing interests

The authors declare that they have no competing interests.

## Authors' contributions

CK and ASR performed the statistical analyses, interpreted the results and CK wrote the manuscript. ASR, GBMM and BMK were involved in the design and data assessment and BMK is the principal investigator of KiGGS. ASR and GBMM were involved in writing the manuscript and made substantial contributions. BMK and RPL revised the manuscript critically. All authors have read and approved the final version.

## Pre-publication history

The pre-publication history for this paper can be accessed here:


